# Officers and Committees, A. V. M. A.

**Published:** 1898-10

**Authors:** 


### A. V. M. A.

### OFFICERS AND COMMITTEES, 1898–1899.

*Officers:* President, A. W. Clement, Baltimore, Md.; Vice-Presidents, Leonard Pearson, Philadelphia, Pa.; A. H. Baker; Chicago, Ill.; S. B. Nelson, Pullman, Washington; Secretary, S. Stewart, 7½ So. James St., Kansas City, Kansas; Treasurer, Wm. Herbert Lowe, Paterson, N. J.

*Executive Committee:* C. A. Cary, Chairman, Auburn, Ala.; D. E. Salmon, Washington, D. C.; J. F. Winchester, Lawrence, Mass.; W. Horace Hoskins, Philadelphia, Pa.; R. R. Bell, New York City, N. Y.; M. H. Reynolds, St. Anthony's Park, Minn.; A. T. Peters, Lincoln, Neb.

*Finance Committee:* C. C. Lyford, 821 Third Ave., S., Minneapolis, Minn.; J. R. Mitchell, Evansville, Ind.; Lemuel Pope, Portsmouth, N. H.

*Committee on Publication:* W. L. Williams, Ithaca, N. Y., R. R. Bell, New York City, N. Y.; Wm. Herbert Lowe, Paterson, N. J.; R. P. Lyman, Hartford, Conn.; S. Stewart, Kansas City, Kan.

*Army Legislation:* D. E. Salmon, Washington, D. C.; M. Stalker, Ames, Iowa; F. H. Mackie, Fair Hill, Md.; W. Horace Hoskins, Philadelphia, Pa.; J. P Turner, 910 O St., N. W., Washington, D. C.

*Committee on Resolutions:* Leonard Pearson, Philadelphia, Pa.; James Law, Ithaca, N. Y.; T. E. White, Columbia, Mo.; J. C. Norton, Phœnix, Arizona; L. A. Merillat, Chicago, Ill.

*Intelligence and Education:* M. Stalker, Chairman, Ames, Iowa; James Law, Ithaca, N. Y.; F. H. Osgood, Boston, Mass.; Leonard Pearson, Philadelphia, Pa.; Joseph Hughes, Chicago, Ill.

*Committee on Diseases:* C. W. Heitzman, 1410 Thalia St., New Orleans, La.; Tait Butler, Starkville, Miss.; H. D. Gill, New York City, N. Y., John M. Parker, Haverhill, Mass.; H. P. Eves, Wilmington, Del.

